# Factors affecting health services strategic purchasing for breast cancer patients: a mixed study in Iran

**DOI:** 10.1186/s12962-021-00324-1

**Published:** 2021-10-18

**Authors:** Samereh Yaghoubian, Mohammad Ali Jahani, Zeynab Farhadi, Ghahraman Mahmoudi

**Affiliations:** 1 Medical and Health Services Administration, Hekmat Hospital, Mazandaran Social Security Organization, Sari, Iran; 2 Social Determinants of Health Research Center, Health Research Institute, Babol University of Medical Sciences, Babol, Iran; 3 Hospital Administration Research Center, Sari Branch, Islamic Azad University, Sari, Iran

**Keywords:** Cancer of breast, Patients, Health services, Purchase

## Abstract

**Background:**

Inappropriate ways of health services purchasing for cancer patients can be challengeable and costly and seriously affect the access to health services and outcomes. This study aimed at Factors affecting health services strategic purchasing for breast cancer patients.

**Methods:**

As a mixed study, this research was conducted in Iran in 2020. In the qualitative phase, 21 specialists and professionals in the field of health services purchasing were purposefully selected and interviewed. After data saturation, interviews were analyzed with the framework analysis and a structured questionnaire was made based on these analyses. 400 breast cancer patients were selected by randomized sampling and completed the questionnaire. Data were analyzed with SPSS_23_ in p < .05.

**Results:**

The highest mean rate of the three main categories belonged to “insurance trusteeship” (4.71 ± .35), followed by “supply management” (4.48 ± .27) and “financial performance” (4.48 ± .37). There were significantly differences between the mean rates of the main categories and the cut-off point (p < .001). In addition, “insurance trusteeship” ranked first (2.58), followed by financial performance (1.77) and supply management (1.65).

**Conclusion:**

Of main components in health services strategic purchasing for breast cancer patients, insurance trusteeship, supply management, and financial performance ranked first to third, respectively. Therefore, healthcare policy-makers should consider the placement of insurance trusteeship and coordinate between purchasers and providers for making reform in the health system.

## Introduction

Breast cancer is the most common cancer among women worldwide [1–3]. With the highest mortality rate among all common cancers, it seriously threatens women’s health [4–6]. Because of increase in the life expectancy, urban growth and following western lifestyle, this cancer is more common among women in developing countries [4, 7], resulting in 50% of breast cancer cases and 58% of reported mortality rate of breast cancer among women in these countries [7]. Due to the epidemiological model similarity of breast cancer in Iran with that of East-Mediterranean and developing countries [8], this cancer incidence is more common among Iranian younger women than that in developing countries [1] and occurs about 10–15 years earlier than that in developed countries and the globe mean [8].

Since the early diagnosis and treatment of cancer can save up costs [9] and enhance patients’ survival [10, 11], the lack of care coordination among various providers [12] and weak payment infrastructures designed by different insurers [13] increase the catastrophic expenditure of disease from %14.2 to %22.2 [14], resulting in omission of follow up, especially by patients with low incomes and more out of pocket [15]. As purchasing has a determinant role in promoting high-quality health services and improving costs [16, 17], the traditional or passive service purchasing process (i.e. assigning a determined budget or payment of invoices with indirect and pre-determined patterns and without any active interaction and communication with benefit packages or providers’ regulations and principles) [18] may result in a barrier to accessing services and changes in disease states [19]. The reason is that such a service has a low financial protection and makes inequity access [20] and may cause the diagnosis of a disease in its progressive stage [8].

Therefore, health service strategic purchasing is identified as one of the key tools for accessing Universal Health Coverage (UHC) and enhancing patients’ access to health services and their financial protection [21]. By actively determining the intervention, providers and the way of services purchasing, it promotes the performance of the health system by effectively allocating financial resources for care providers based on their quality and efficiency [22].

Ibe and colleagues showed in their study that purchasers (insurers) as public representations for health service purchasing use effective mechanisms for determining and reflecting public needs and priorities that results in monitoring the communication process between themselves and providers as well as by contacting and the payment mechanisms [23]. Aroh and colleagues revealed in their study that strategic purchasing has changed into a framework for the purchasers of health care in order to consider the high-quality and affordable service delivery [24]. However, Hagenaars and colleagues showed in their study that more active purchasing patterns in health systems make them more complex and can increase administrative burden [25].

The evidence shows that passive purchasing is common in Iran [19]. In spite of financing a part of some disease costs, including cancers by the government from the governmental annual budget, the catastrophic health expenditures has been increased considerably in nationwide. Since the annual budget is defined and fixed, payment to providers is based on predetermined patterns and without active interaction with them and service packages [14, 19]. Therefore, by increasing the catastrophic expenditure of disease and imposing it on patients [18], the Universal Health Coverage (UHC) is impossible without protecting people from disease-related financial problems [14]. For more emphasis on the influence of strategic purchasing on the health outcome, it is needed that some reforms are placed in the health system, especially service delivery [26], purchasers and financial function [27], and increasing efficiency and accountability for people [23]. This can guarantee service accessibility and its affordability [15] and decrease unnecessary treatments or services as well as increasing in the quality of care [28]. As a result, this study aimed at Factors affecting health services strategic purchasing for breast cancer patients and establishing a decision-making base for Iranian health system policy-maker.

## Methods

Conducted in 2020, this study had a mixed method approach and was designed in three steps. In step one, the related literature was searched with the keywords such as “strategic purchasing”, “health services” and “breast cancer”. The retrieved literature constructed the theoretical base of questions designed for interviewing. Some questions were included on interviewees’ information, motivations, experiences, values, desires and opinions regarding health service purchasing for cancer patients.

In step two, as the qualitative phase (for data gathering) a semi-structured and in-depth interview was programmed. For accessing ones with more information on the topic, a snow-ball sampling method was applied and interviews were done until data saturation. 21 specialists and professionals in the field of health service strategic purchasing were selected purposefully and interviewed. All participants held executive positions in the health system and they were in two disciplines: clinical and basic sciences. Clinical disciplines include general practitioners, specialists and pharmacists and the basic sciences included in health policy, health services management, epidemiology and social medicine. Most interviews were conducted face-to-face; only two of them were done by phone. Interviews were revised several times and codified by labeling them and identifying main categories and themes/sub-themes (Table 1).

**Table 1:** The main categories and themes/sub-themes involved in the strategic purchasing needed for health services for breast cancer patients in Iran in 2020

Main category	Themes/sub-themes
Supply management	Strategic purchasing infrastructures (Scientific and cultural infrastructure/Information infrastructure/Political and social infrastructure/Economic infrastructure/Legal infrastructure/Policy and decision making infrastructure)
	Practical guidelines (Therapeutic guidelines/Insurance guidelines)
	Trusteeship structure (Ministry of Health as a policy maker and health system supervisor/Providers independence from trusteeship/financiers Independence from trusteeship/Decentralization in health system)
	Service package (Definition of special patients groups/Definition of type of service/Designing benefits package (smaller and basic Package)/Level of services/Payment system/Purchase of specialized services)
	Service quality (Use of new technology/Infrastructures of human resources, equipment and special care/ranking of care quality and selection of top providers)
	Service quantity (services availability/Access to services)
	Role of other organizations and groups (Use of the capacity of NGOs to cover supporting and supplementary services at levels two and three/The role of the medical system organization in managing and contracting with the private sector/The role of insurance companies in the purchase of complementary services)
	Training (Training and strengthening providers, producers, policymakers’ knowledge on the insurance)/Patients education/Training service purchasers (Awareness of new therapies)
Insurance trusteeship	Establishment of an insurance thinking (Integrated and coordinated insurance policies (resources, structure, payment model)/Defining insurance identity for providers/Changing the Providers attitudes (insurances are not fund, differentiating between supportive thinking and insurance)/Changing the therapeutic' s approaches to the health perspective (prevention system and screening)/Changing the conflicting policies and personal interests of decision-makers)
	Strategic management (Managerial determination to execute strategic purchasing/Purchase prioritization based on needs/Process modification/Contract management/Strong control system/Creating Competition (by using the motivational issues)/Necessity of managing the insurance system by non-physicians)
	Communication (Internal communication (between purchasers and importers of equipment and pharmaceuticals, etc.)/International communications/Cross-sector collaboration (between purchasers and supplier))
Financial performance	Price (Setting the services price (Service price correct)/Cost classification/Bargaining power and mass purchasing)
	Efficiency and effectiveness (Economic assessment/Dissimilar payment to different providers)
	Resource provision (Investment (income generation)/resource pooling Proper purchase (optimal allocation of fund)/Expert human resources/care Management)

In step three, for Factors affecting health services strategic purchasing for breast cancer patients, a 61-item structured questionnaire was made based on main categories and themes/subthemes. The questionnaire involved three parts. The first part included information on patients’ demographic variables (such as age, educational level, marriage status, occupation, being insurance). The second part consisted of some questions on risk factors of disease etiology, the relationship between these risk factors and health services, prevention ability, follow up and early diagnosis of the disease and the history of patients’ health. In the third part of questionnaire, some special questions were included based on the main categories of the study (supply management, insurance trusteeship and financial performance) as well as the five levels of health services (i.e. prevention, diagnosis, treatment, rehabilitation, and palliative care needed by the patients) in a 5-pointed Likert type scale ranging from the minimum = 1 to the maximum = 5. The questionnaire content validity was confirmed by some specialists and its reliability (internal consistency) amounted to a = 0.894in a pre-test on 30 patients.

The research population included all patients with breast cancer referred to the hospital for treatment after certain diagnosis. The classification of patients was based on service provider centers in Iran. Of them, 400 women were selected [29] with randomized sampling as the research sample and participated by consent in the study. They were informed that their information would remain confident. SPSS 23 was used for data analysis and Kolmogorov–Smirnov test was used to determine the distribution of samples.

One-sample t-student test and ANOVA were used by comparing a mean score of each component in the research population with an assumed mean score. The cut-point was assumed over 70% of mean rates and over 3.5 in a 5-pointed Likert type scale (In the 5-point Likert scale, we considered scores 3 and 4 as the minimum acceptable score (average = 3) (good = 4) and the mean of these two scores as the cut-off point). Friedman Test was used for ranking the components.

## Results

Findings of the study in the qualitative phase showed that the main categories and themes/sub-themes for strategic purchasing of health services for breast cancer patients can be categorized in three main axes and several main themes and some small items. The main components include supply management, insurance trusteeship, and financial performance (Table 1).

The descriptive demographic data of patients with breast cancer were depicted in Table 2 based on the main axes of the strategic purchasing of health services. The majority of the patients (180, 45%) were in the age range of 41–50 years old. 277 patients (69.2%) had no university educational degrees. The Body Mass Indexes (BMI) of 293 patients (73.2%) were in normal range or overweight. Table 2 shows the demographic and qualitative variables.

**Table 2:** The distribution of variables of health service strategic purchasing and demographic variables description of breast cancer patients in Iran in 2020

Variables	Frequency (%)	Supply management		Insurance trusteeship		Financial performance	
		Mean ± SD	p-value	Mean ± SD	p-value	Mean ± SD	p-value
Age range							
> 40	23.5% (94)	.22 ± 4.71	< .01	.30 ± 4.74	.01	.36 ± 4.51	.40
41–50	45% (180)	.29 ± 4.60		.39 ± 4.64		.39 ± 4.45	
< 50	31.5% ( 126)	.29 ± 4.58		.30 ± 4.75		.36 ± 4.48	
BMI							
< 18.5	26.8% (107)	.08 ± 4.82	< .01	.30 ± 4.84	< .01	.50 ± 4.75	< .01
18.5–25	37.3% ( 149)	.26 ± 4.69		.25 ± 4.82		.35 ± 4.57	
< 25	35.9% ( 144)	.28 ± 4.59		.37 ± 4.65		.37 ± 4.44	
Habitation							
City	64.2% ( 257)	.28 ± 4.61	.47	.35 ± 4.69	.27	.37 ± 4.45	.06
Village	35.8% (143)	.28 ± 4.63		.35 ± 4.73		.37 ± 4.52	
Marriage							
Married	91.0% (364)	.29 ± 4.62	.87	.34 ± 4.71	.22	.36 ± 4.48	.60
Single	9.0% (36)	.21 ± 4.61		.41 ± 4.63		.47 ± 4.44	
No. of children							
0	7.3% (29)	.23 ± 4.62	.01	.44 ± 4.62	.41	.49 ± 4.45	.81
1–2	58.2% (233)	.25 ± 4.65		.33 ± 4.71		.35 ± 4.49	
≤ 2	34.5% (138)	.32 ± 4.56		.36 ± 4.70		.38 ± 4.47	
Education							
Non-university	69.2% (277)	.28 ± 4.62	.99	.34 ± 4.72	.09	.35 ± 4.47	.57
University	30.8% (123)	.27 ± 4.62		.37 ± 4.66		.43 ± 4.49	
Insurance							
Yes	95.5% (382)	.28 ± 4.63	< .01	.34 ± 4.71	.17	.37 ± 4.48	.16
No	4.5% (18)	.26 ± 4.45		.40 ± 4.59		.35 ± 4.35	

As Table 3 shows, the highest mean rate of the three main categories belonged to “insurance trusteeship” (4.705 ± 0.35). As can be seen, there were significantly differences between the mean rates of the main categories and the cut-off point (= 3.5) (p < .001).

**Table 3:** T-test results for comparing the mean rates of the main categories of health service strategic purchasing for breast cancer patients in Iran in 2020

Components	Test value = 3.5					
	Mean ± SD	t	p-value	Mean difference	95% confidence interval of the difference	
					Lower	Upper
Supply management	4.484 ± .27	70.88	< .001	.98	.95	1.01
Strategic purchasing infrastructures	4.60 ± .56	39.29	< .001	1.10	1.05	1.16
Practical guidelines	4.70 ± .44	54.74	< .001	1.20	1.16	1.25
Trusteeship structure	4.31 ± .63	25.86	< .001	.81	.75	.87
Service package	4.72 ± .31	77.53	< .001	1.22	1.19	1.25
Service quality	3.62 ± .27	8.81	< .001	.12	.09	.14
Service quantity	4.54 ± .44	46.92	< .001	1.04	1	1.09
Role of other organizations and groups	4.50 ± .52	37.92	< .001	1	.94	1.05
Training	4.64 ± .45	50.23	< .001	1.14	1.09	1.18
Insurance trusteeship	4.705 ± .35	68.51	< .001	1.20	1.17	1.23
Establishment of an insurance thinking	4.70 ± .41	58.70	< .001	1.20	1.16	1.24
Strategic management	4.72 ± .35	68.71	< .001	1.22	1.19	1.26
Communication	4.66 ± .46	50.36	< .001	1.16	1.11	1.26
Financial performance	4.481 ± .37	52.11	< .001	.98	.94	1.01
Price	4.16 ± .59	22.43	< .001	.66	.61	.72
Efficiency and effectiveness	4.68 ± .41	57.74	< .001	1.18	1.14	1.22
Resource provision	4.66 ± .41	56.88	< .001	1.16	1.12	1.20

Table 4 shows the results of Friedman Test for ranking the categories and themes of strategic purchasing of health services for breast cancer patients. After standardization of the components, it was revealed that “insurance trusteeship” ranked first (mean rank = 2.58), followed by “financial performance” (mean rank = 1.77) and “supply management” (mean rank = 1.65). The first rank of main themes in the category of “insurance trusteeship” belonged to the “strategic management” (mean rank = 3.00).

**Table 4:** Ranking the main categories/themes of health service strategic purchasing for breast cancer patients in Iran in 2020

Main categories	Mean rank	Main themes	Mean Rank
Supply management	1.65	Strategic purchasing infrastructures	3.52
		Practical guidelines	1.04
		Trusteeship structure	5.60
		Service package	8.00
		Service quality	4.10
		Service quantity	7.00
		Role of other organizations and groups	3.25
		Training	3.49
Insurance trusteeship	2.58	Establishment of an insurance thinking	2.00
		Strategic Management	3.00
		Communication	1.01
Financial performance	1.77	Price	2.19
		Efficiency and effectiveness	1.02
		Resource provision	2.80

## Discussion

The results showed that “insurance trusteeship” has the highest mean rate regarding the strategic purchasing of health services for breast cancer patients. This clearly shows the importance of insurance trusteeship and its related themes among breast cancer patients. Tangcharoensathien and colleagues stated in their research that the strategic purchasing is key to the Universal Health Coverage (UHC) policy that can be effective in better and equity access to the primary health care and financial risk protection, if the purchasers managed it properly. They emphasized the vital role of the strategic purchasing in supporting patients’ appropriate referral to the secondary and tertiary levels [21]. Moreover, purchasers can manage the activities of service providers by regulating contractions and consequent promotion of service quality as well as motivating competition between them. Therefore, services delivery is advanced through decrease in costs and increase in efficiency as well as achieving organizational flexibility and making accountability to patients’ needs [30].

Patcharanarumol and colleagues state in their study that although purchasers should have some clear mechanisms in determining and reflecting people’s needs, they have no clear mechanisms for designing benefit packages and purchase based on the opinions of their own consultative and technical committees and patients directly are referred to hospitals and professional health providers. Therefore, strategic purchasing needs a certain logical framework and insurers’ direction in order to ensure that public health priorities are relevant to the decision-making on purchasing and purchasers’ organizational capacities are well applied [31]. As a result, the insurance trusteeship is more important for cancer patients because of their encounter with challenges such as high costs for treatment, intensive care, considerable gap between cancer prevention and treatment, inappropriate access to protective and treatment services, limited capacity of public providers and long waiting time for receiving related services. These factors motivate cancer patients to continuously search for service types and purchasing manners and select for the best providers with high quality, low cost services. Therefore, patients demand insurers’ active involvement in health service purchasing.

Findings also showed that there were significant differences between the main categories of strategic purchasing of health services for breast cancer patients (Compared to the vector 3.5). This manifests the importance of these purchasing component.

As stated by Chen and colleagues, strategic purchasing makes a close work relationship between suppliers, providers and purchasers in a way that developing and deepening this relationship in a long-term provides mutual benefits for all parties (including the advantage of sustainable competition among suppliers and providers and a powerful and beneficial structure in the purchasing organization as well as valuable services for customers [32]. Therefore, in line with the study by Chen and colleagues, this study emphasized the relation between strategic purchasing, supply management, customer accountability and financial performance.

However, Sanderson and colleagues showed in their study that despite their emphasis on supply management and purchasing regulations as great opportunities for the health system reform, strategic purchasing theories focus on challenges and weaknesses of purchasing and ignores the improvement of purchasing performance [33]. In addition to considering known components, strategic purchasing depends heavily on coordinating all possible relations between all stakeholders, such as patients, purchasers, providers and suppliers and trying to achieve efficient performance by making them to be compatible. For instance, managing the providers as services leaders can result in training and empowering patients for better health management, selecting correct treatment options, and patient–provider strategic cooperation through a shared decision-making mechanism. In other words, purchasers can focus on the output of purchasing process through active interaction with benefit packages or suppliers’ regulations and promote their quality and effectiveness by emphasizing the reformative innovations such as ranking providers. This results in patients’ correct familiarity with services, as they do not recognize correct services due to their low medical knowledge or asymmetry of information.

In this study, the main category “insurance trusteeship” and its main theme “strategic management” ranked first from views of breast cancer patients. Munge and colleagues showed in their study that Universal Health Coverage (UHC) in low and Middle Income Countries (LMICs) encounter several challenges such as health-related impoverishment in reaching high-quality services. They concluded that for implementation of strategic purchasing mechanism, purchasing organizations need regulatory and managerial infrastructures for prioritizing and determining the purchasing efficacy and allocating resource and developing and managing contractions, embedded in the structural and organizational specifications of insurance organizations [34]. So, it is needed that the role of insurers as well as their strategic management approaches is emphasized.

Witter and colleagues showed in their study that the main functions of strategic purchasing are related to the role of the government, patients and providers and an insurance organization effectively intervenes in the health system and regulating supply-side structures by managing contractions, financial support of services based on outputs/outcomes and consequent high-quality services [35]. However, Nejad and colleagues stated in their study that if there is no unique defined organization as insurance trusteeship for regulating, organizing and managing purchasers, roles, duties, tasks and regulations for purchasing and intervention cannot be determined. This results in fragmentation, wastage of resources, parallelism and conflict of interests and consequently, no access to possible advantages of the strategic purchasing of health services [36]. A powerful insurance trusteeship in health system with a monopoly power and better bargaining capacity with service providers about service quality and prices and enough knowledge on patients’ needs can satisfy the main aim of purchasing (i.e. making relationship between pooled resources and effective services), resulting in an effective and efficient and integrative health system.

## Limitations of the study

Limitations of this study in the qualitative phase include: legal and regulatory restrictions, lack of right of choice, the accuracy of the researcher’s interpretations and inability to generalize the results to all diseases or conditions. Limitations of the study in the quantitative phase include setting unenforceable cooperation conditions, unavailability of some identified patients, and inadequate general conditions of some patients.

## Conclusion

This study showed that among main components of the strategic purchasing of health services for breast cancer patients, insurance trusteeship has the first rank, followed by financial performance and supply management. This shows that insurance trusteeship is more important than health services financing and comprehensive reforms can be done in the health system by help of insurers and their potentialities such as the strategic management.

Purchasers can program to actively select the best providers and high-quality services by strategic purchasing and comprehensive management and monitor patients' needs and exactly define benefit packages. These help them to ensure patients' fair access to needed services through motivating various providers to be dynamic and competitive. It promotes efficacy and stability of resources and accessing high efficiency and resource sustainability. Governmental policy-makers in the health system can ensure the better health services by improving the placement of insurance trusteeship as a unique active purchaser of health system and coordinating purchaser-provider affairs and regulating the supplier-side. They need to delegate a part of their tasks and decentralize the healthcare system.

## Data Availability

The authors have full control over the primary data. The data analyzed in this study are housed at hospital administration research center in Islamic Azad University, Sari Branch, 7 km Sea Road, Sari, Mazandaran, Iran. According to the ethical committee approval, this dataset is subject to ethical restrictions and local data protection regulations regarding qualitative raw data, since participant privacy could be compromised. Participants did not consent to have their full transcripts made available for third parties. All relevant data for the conclusions are presented in the manuscript.

## Abbreviations

CASP: Critical Appraisal Skills Program
CVI: Content validity index
CVR: Content validity ratio
KMO: Kaiser–Meyer–Olkin
MSC: Master of Science
UHC: Universal Health Coverage
WHO: World Health Organization
